# H-Theorem in an Isolated Quantum Harmonic Oscillator

**DOI:** 10.3390/e24081163

**Published:** 2022-08-20

**Authors:** Che-Hsiu Hsueh, Chi-Ho Cheng, Tzyy-Leng Horng, Wen-Chin Wu

**Affiliations:** 1 Department of Optoelectric Physics, Chinese Culture University, Taipei 111, Taiwan; 2 Department of Physics, National Taiwan Normal University, Taipei 106, Taiwan; 3 Department of Physics, National Changhua University of Education, Changhua 500, Taiwan; 4 Department of Applied Mathematics, Feng Chia University, Taichung 407, Taiwan

**Keywords:** quantum harmonic oscillator, H-theorem, thermalization, Shannon entropy, barrier potential, decoherence

## Abstract

We consider the H-theorem in an isolated quantum harmonic oscillator through the time-dependent Schrödinger equation. The effect of potential in producing entropy is investigated in detail, and we found that including a barrier potential into a harmonic trap would lead to the thermalization of the system, while a harmonic trap alone would not thermalize the system. During thermalization, Shannon entropy increases, which shows that a microscopic quantum system still obeys the macroscopic thermodynamics law. Meanwhile, initial coherent mechanical energy transforms to incoherent thermal energy during thermalization, which exhibiting the decoherence of an oscillating wave packet featured by a large decreasing of autocorrelation length. When reaching thermal equilibrium, the wave packet comes to a halt, with the density distributions both in position and momentum spaces well-fitted by a microcanonical ensemble of statistical mechanics.

## 1. Introduction

H-theorem plays an important role in the time evolution of system in both the classical and quantum statistical mechanics. In particular, it identifies the entropy function appearing in the second law of thermodynamics. It is a longstanding question on the microscopic description of the second law of thermodynamics. One fundamental issue is whether an isolated system can reach thermal equilibrium, i.e., the state with maximum entropy [1,2,3,4,5,6,7,8]. In isolated quantum systems, it is especially important to see how the reversible microscopic quantum mechanics conceals the irreversible macroscopic phenomena of thermodynamics. Therefore, a directional time evolution of isolated quantum systems at nonequilibrium would often reveal the thermalization process concealed in quantum mechanics. Fortunately, ultracold quantum gases, which are pure and controllable, provide an excellent platform to study the nonequilibrium dynamics for isolated quantum systems. In atomic Bose–Einstein condensate (BEC) experiments, Kinoshita et al. [9] showed no evidence of thermalization by pairwise collision, from the Tonks–Girardeau limit to the intermediate coupling regime. Nevertheless, the dissipative motion of oscillating BEC in a disorder trap, first studied by Dries et al. [10] and further investigated by Hsueh et al. [11], does manifest the thermalization of an isolated quantum system. In particular, the noninteracting-limit case has been numerically studied in [12], which depicts Shannon entropy increasing to its maximum during thermalization, and at equilibrium, the initial mechanical energy is transformed to the thermal energy consisting of evenly distributed kinetic and potential energies.

In equilibrium statistical mechanics, one primary task is to determine the distribution function. Owing to the dependence of interaction, exact distribution function of a many-body system is usually not accessible. In nonequilibrium statistical mechanics, in contrast, the most difficult task is to determine the governing factors that describe the thermalization. It will be shown that the issue is not related to whether there is another new law describing the thermalization, but on how the governing microscopic laws can properly describe it and at the same time obey macroscopic thermodynamic laws. In this paper, we try to answer these two questions by studying an isolated quantum harmonic oscillator (QHO) that thermalizes through a single barrier. The disorder trap used for thermalization in [12] is constituted by a harmonic trap superposed with a Gaussian correlated disorder potential, which consists of many bumps in various sizes. Here, we demonstrate that a harmonic trap superposed with a single bump barrier alone can thermalize QHO as well through wave scattering. In addition, in [12], thermalization starts from a mobilized ground state of harmonic trap, while in this paper, besides a ground state of harmonic trap, we additionally employed a mixture of two pure states (such as ground and first excited states) of harmonic trap as the initial condition in order to exhibit different entropy evolution behaviors during thermalization. Moreover, here we presented a rigorous theoretical derivation of entropy production that shows that additional barrier potential can cause the increasing of entropy and leading to thermalization, while harmonic trap alone would not.

To be more specific, we studied this thermalization process by the time evolution of density distribution, for which we have numerically solved the time-dependent Schrödinger equation (TDSE), Equation (8), with initial condition, Equation (17), with ℏω and the harmonic trapping length $l=\sqrt{\hbar /m\omega }$ taken as the units of energy and length. To numerically integrate Equation (8), we use the method of lines with spatial discretization by a Fourier pseudospectral method and time integration by an RK45 scheme [13].

In this study, we will show that, after being released from the proposed initial coherent state with an excessive potential energy by placing the wave packet away from the center of trap initially, the wave packet would oscillate in this trap, wave-scattered by the single bump barrier located in the center of trap, and at the same time gradually transform the initial excessive potential energy into incoherent thermal energy. The whole phenomenon is analogous to a classical damped harmonic oscillator with the single bump barrier acting as the friction. When evolution time is long enough ($t\gg t_{\mathrm{th}}$ with $t_{\mathrm{th}}$ being the thermalization time), the system approaches the equilibrium. During the relaxation to its equilibrium, we found that an exchange between kinetic energy and potential energy continues to happen but with diminishing flux. In addition, we observed that the thermalization process of such a microscopic quantum system would still obey the second law of thermodynamics by the examination of increasing Shannon entropy. When equilibrium is reached, the oscillation stops, kinetic-potential energy exchange comes to a halt, Shannon entropy reaches its maximum, and the initial excessive potential energy is totally dissipated into incoherent thermal energy.

The paper is organized as follows: in Section 2, Shannon entropy in momentum space is adopted here to describe the thermalization of the current isolated quantum system, and an entropy production equation in momentum space is derived to analyze the effect of external potential. In Section 3, we found that harmonic trap alone will not generate any entropy, but with a barrier potential; in addition, entropy can then be produced, which means that the recruitment of a barrier potential into the harmonic trap would cause thermalization. In addition, the entropy production during thermalization is accompanied by a decoherence of wave packet featured by a large decreasing of autocorrelation length. In Section 4, when reaching thermal equilibrium eventually, we show that the density distributions both in position and momentum spaces are well fitted by a microcanonical ensemble of statistical mechanics. In Section 5, we study the time evolution of energy and investigate how different, from a pure QHO kinetic and potential, energies exchange with each other during thermalization, which describes how a coherent mechanical energy transforms to an incoherent thermal energy. Finally, we present the conclusions in Section 6.

## 2. Model and Methods

In quantum statistics, one often follows the von Neumann entropy:

(1) $$S_{v}\left(t\right)=-k_{B}\mathrm{Tr}\left[\hat{\rho }\left(t\right)\ln\hat{\rho }\left(t\right)\right],$$

with $\hat{\rho }$ being the density operator. The production rate of $S_{v}$ is then

(2) $$\frac{dS_{v}\left(t\right)}{dt}=-k_{B}\mathrm{Tr}\left[\frac{\partial \hat{\rho }\left(t\right)}{\partial t}\ln\hat{\rho }\left(t\right)+\frac{\partial \hat{\rho }\left(t\right)}{\partial t}\right].$$

The second term in Equation (2) vanishes due to the normalization of the total number of states. It is a general concept that $S_{v}\left(t\right)$ as well as $dS_{v}\left(t\right)/dt$ are basis independent. In terms of an arbitrary basis |i〉,

(3) $$\frac{dS_{v}\left(t\right)}{dt}=-k_{B}\sum_{i,j}\langle i|\frac{\partial \hat{\rho }}{\partial t}|j\rangle \langle j|\ln\hat{\rho }|i\rangle .$$

However, if the basis |i〉 is chosen to be eigenkets of $\hat{\rho }$ or energy eigenkets, then

(4) $$\frac{dS_{v}\left(t\right)}{dt}=-k_{B}\sum_{i,j}\langle i|\frac{\partial \hat{\rho }}{\partial t}|j\rangle \langle j|\ln\hat{\rho }|i\rangle =\frac{ik_{B}}{\hbar }\sum_{i,j}\langle i|\left[\hat{H},\hat{\rho }\right]|j\rangle \langle j|\ln\hat{\rho }|i\rangle =0.$$

Here, $\hat{H}$ is the Hamiltonian. The vanishing of $dS_{v}\left(t\right)/dt$ in Equation (4) indicates that von Neumann entropy can not offer any entropy production for an isolated quantum system going through thermalization [14,15].

Alternatively, one can consider the Shannon entropy ($S_{Q}$) whose production rate is defined as [16,17,18]

(5) $$\frac{dS_{Q}\left(t\right)}{dt}=-k_{B}\sum_{i}\langle i|\frac{\partial \hat{\rho }}{\partial t}\left|i\rangle \ln\langle i\right|\hat{\rho }|i\rangle =\frac{ik_{B}}{\hbar }\sum_{i}\langle i|\left[\hat{H},\hat{\rho }\right]|i\rangle \langle i|\ln\hat{\rho }|i\rangle ,$$

where the basis |i〉 is the eigenkets of operator $\hat{Q}$, which is arbitrary except for using the eigenkets of either $\hat{\rho }$ or $\hat{H}$. In addition, the basis |i〉 is not suitable to be chosen as the eigenkets of other complete sets of commuting observables (CSCO). In general, position $\hat{x}$ and momentum $\hat{p}$ are suitable choices of $\hat{Q}$ since they are not both CSCO. When the basis |i〉 is chosen to be momentum eigenkets, the corresponding Shannon entropy and its production rate are

(6) $$S_{p}\left(t\right)=-\frac{k_{B}}{2\pi }\int_{-\infty }^{\infty }\rho_{kk}\left(t\right)\ln\left[\frac{\rho_{kk}\left(t\right)}{2\pi l}\right]dk,$$

and

(7) $$\frac{dS_{p}\left(t\right)}{dt}=-\frac{k_{B}}{2\pi }\int_{-\infty }^{\infty }\frac{\partial \rho_{kk}\left(t\right)}{\partial t}\ln\left[\frac{\rho_{kk}\left(t\right)}{2\pi l}\right]dk,$$

where $\rho_{kk}\equiv {\left|\tilde{\psi }\left(k,t\right)\right|}^{2}$ is the momentum distribution with $\tilde{\psi }\left(k,t\right)=\langle k|\psi \left(t\right)\rangle$.

In this paper, we focus on a one-dimensional quantum harmonic oscillator, initially released from an off-center position, that corresponds to an out-of-equilibrium state [10,19,20,21]. The system is well described by the time-dependent Schrödinger equation (TDSE)

(8) $$i\hbar \frac{\partial \psi \left(x,t\right)}{\partial t}=\left[-\frac{\hbar^{2}}{2m}\frac{\partial^{2}}{\partial x^{2}}+V\left(x\right)\right]\psi \left(x,t\right),$$

where *m* is the particle mass, and the external potential is $V\left(x\right)=V_{0}\left(x\right)+V_{1}\left(x\right)$ consisting of a confining harmonic well $V_{0}\left(x\right)=m\omega^{2}x^{2}/2$ with ω the trapping frequency and a barrier potential $V_{1}\left(x\right)$ described later.

The real-space TDSE, Equation (8), can be Fourier transformed to momentum space,

(9) $$i\hbar \frac{\partial \tilde{\psi }\left(k,t\right)}{\partial t}=\frac{\hbar^{2}k^{2}}{2m}\tilde{\psi }\left(k,t\right)+\frac{1}{2\pi }\int_{-\infty }^{\infty }\tilde{V}\left(k-k_{1}\right)\tilde{\psi }\left(k_{1},t\right)dk_{1},$$

where $\tilde{\psi }\left(k,t\right)$ and $\tilde{V}\left(k\right)$ are respectively Fourier transforms of wave function ψ(x,t) and external potential V(x). The Fourier transform of harmonic well $V_{0}\left(x\right)$ is

(10) $$\begin{array}{ccc} {\tilde{V}}_{0}\left(k\right) & = & \int_{-\infty }^{\infty }V_{0}\left(x\right)\exp\left(-ikx\right)dx=\frac{m\omega^{2}}{2}\int_{-\infty }^{\infty }x^{2}\exp\left(-ikx\right)dx\\ & = & \frac{m\omega^{2}}{2{\left(-i\right)}^{2}}\int_{-\infty }^{\infty }\frac{\partial^{2}\exp\left(-ikx\right)}{\partial k^{2}}dx=-\frac{m\omega^{2}}{2}\frac{\partial^{2}}{\partial k^{2}}\int_{-\infty }^{\infty }\exp\left(-ikx\right)dx\\ & = & -\pi m\omega^{2}\frac{\partial^{2}\delta \left(k\right)}{\partial k^{2}}, \end{array}$$

where $\delta \left(k\right)=1/\left(2\pi \right)\int_{-\infty }^{\infty }\exp\left(-ikx\right)dx$ is the Dirac delta function. The complex-conjugate of Equation (9) is

(11) $$-i\hbar \frac{\partial {\tilde{\psi }}^{*}\left(k,t\right)}{\partial t}=\frac{\hbar^{2}k^{2}}{2m}{\tilde{\psi }}^{*}\left(k,t\right)+\frac{1}{2\pi }\int_{-\infty }^{\infty }{\tilde{V}}^{*}\left(k-k_{1}\right){\tilde{\psi }}^{*}\left(k_{1},t\right)dk_{1}.$$

By ${\tilde{\psi }}^{*}\left(k,t\right)\times$ Equation (9)$-\tilde{\psi }\left(k,t\right)\times$ Equation (11), we obtain

(12) $$\begin{array}{ccc} i\hbar \frac{\partial \rho_{kk}\left(t\right)}{\partial t} & = & i\hbar \left[{\tilde{\psi }}^{*}\left(k,t\right)\frac{\partial \tilde{\psi }\left(k,t\right)}{\partial t}+\tilde{\psi }\left(k,t\right)\frac{\partial {\tilde{\psi }}^{*}\left(k,t\right)}{\partial t}\right]\\ \; & = & \frac{1}{2\pi }\int_{-\infty }^{\infty }\left[\tilde{V}\left(k-k_{1}\right){\tilde{\psi }}^{*}\left(k,t\right)\tilde{\psi }\left(k_{1},t\right)-{\tilde{V}}^{*}\left(k-k_{1}\right)\tilde{\psi }\left(k,t\right){\tilde{\psi }}^{*}\left(k_{1},t\right)\right]dk_{1}, \end{array}$$

describing the evolution of momentum distribution $\rho_{kk}\left(t\right)$. By $\tilde{V}={\tilde{V}}_{0}+{\tilde{V}}_{1}$, Equation (12) can be reorganized as

(13) $$\begin{array}{ccc} \frac{\partial \rho_{kk}\left(t\right)}{\partial t} & = & -\frac{1}{\pi \hbar }\int_{-\infty }^{\infty }ℑ\left[{\tilde{V}}^{*}\left(k-k_{1}\right)\rho_{kk_{1}}\right]dk_{1}\\ & = & -\frac{1}{\pi \hbar }\int_{-\infty }^{\infty }ℑ\left[{\tilde{V}}_{0}^{*}\left(k-k_{1}\right)\rho_{kk_{1}}\right]dk_{1}-\frac{1}{\pi \hbar }\int_{-\infty }^{\infty }ℑ\left[{\tilde{V}}_{1}^{*}\left(k-k_{1}\right)\rho_{kk_{1}}\right]dk_{1}\\ & = & \frac{\omega }{l^{2}}\int_{-\infty }^{\infty }ℑ\left[\frac{\partial^{2}\delta \left(k-k_{1}\right)}{\partial k_{1}^{2}}\tilde{\psi }\left(k,t\right){\tilde{\psi }}^{*}\left(k_{1},t\right)\right]dk_{1}-\frac{1}{\pi \hbar }\int_{-\infty }^{\infty }ℑ\left[{\tilde{V}}_{1}^{*}\left(k-k_{1}\right)\rho_{kk_{1}}\right]dk_{1}\\ & = & \frac{\omega }{l^{2}}\int_{-\infty }^{\infty }ℑ\left[\delta \left(k-k_{1}\right)\tilde{\psi }\left(k,t\right)\frac{\partial^{2}{\tilde{\psi }}^{*}\left(k_{1},t\right)}{\partial k_{1}^{2}}\right]dk_{1}-\frac{1}{\pi \hbar }\int_{-\infty }^{\infty }ℑ\left[{\tilde{V}}_{1}^{*}\left(k-k_{1}\right)\rho_{kk_{1}}\right]dk_{1}\\ & = & \frac{\omega }{l^{2}}ℑ\left[\tilde{\psi }\left(k,t\right)\frac{\partial^{2}{\tilde{\psi }}^{*}\left(k,t\right)}{\partial k^{2}}\right]-\frac{1}{\pi \hbar }\int_{-\infty }^{\infty }ℑ\left[{\tilde{V}}_{1}^{*}\left(k-k_{1}\right)\rho_{kk_{1}}\right]dk_{1}, \end{array}$$

where $l=\sqrt{\hbar /m\omega }$ (harmonic trapping length), $\rho_{kq}\equiv \tilde{\psi }\left(k,t\right){\tilde{\psi }}^{*}\left(q,t\right)$, and ℑ[A] denotes the imaginary part of *A*. The first term of the right-hand side of Equation (13), which comes from the harmonic potential, indicates the contribution of diagonal elements of the density matrix, while the second term, from the barrier potential $V_{1}$, indicates the contribution of off-diagonal elements, which could lead to the decoherence of a density matrix.

Substitution of Equation (13) into Equation (7) yields

(14) $$\begin{array}{ccc} \frac{dS_{p}\left(t\right)}{dt} & = & \frac{k_{B}}{2\pi^{2}\hbar }\int_{-\infty }^{\infty }\int_{-\infty }^{\infty }ℑ\left[{\tilde{V}}^{*}\left(k-k_{1}\right)\rho_{kk_{1}}\right]\ln\left(\frac{\rho_{kk}}{2\pi l}\right)dkdk_{1}\\ & = & -\frac{k_{B}\omega }{2\pi l^{2}}\int_{-\infty }^{\infty }ℑ\left[\tilde{\psi }\left(k,t\right)\frac{\partial^{2}{\tilde{\psi }}^{*}\left(k,t\right)}{\partial k^{2}}\right]\ln\left(\frac{\rho_{kk}}{2\pi l}\right)dk\\ & + & \frac{k_{B}}{2\pi^{2}\hbar }\int_{-\infty }^{\infty }\int_{-\infty }^{\infty }ℑ\left[{\tilde{V}}_{1}^{*}\left(k-k_{1}\right)\rho_{kk_{1}}\right]\ln\left(\frac{\rho_{kk}}{2\pi l}\right)dkdk_{1}. \end{array}$$

It will be shown later that only the second term of the right-hand side of the equation above, coming from barrier potential $V_{1}$, would contribute to the increasing of entropy.

For QHO, the generalized coherent states (GCSs) |n,α〉, with *n* denoting the quantum number and α the complex quantum number, have the following forms in position and momentum spaces [22]:

(15) $$\begin{array}{c} \varphi_{\alpha ,n}\left(x,t\right)=\langle x|\alpha ,n\rangle =\frac{1}{\sqrt{l}}\phi_{n}\left(\frac{x-\bar{x}}{l}\right)\exp\left(-in\omega t\right)\exp\left[-i\left(\frac{\omega t}{2}-x\bar{k}+\frac{\bar{x}\bar{k}}{2}\right)\right], \end{array}$$

(16) $$\begin{array}{c} {\tilde{\varphi }}_{\alpha ,n}\left(k,t\right)=\langle k|\alpha ,n\rangle =i^{n}\sqrt{2\pi l}\phi_{n}\left[l(k-\bar{k})\right]\exp\left(-in\omega t\right)\exp\left[-i\left(\frac{\omega t}{2}-\bar{x}k+\frac{\bar{x}\bar{k}}{2}\right)\right] \end{array}$$

with $\bar{x}\left(t\right)/l=\sqrt{2}\alpha \cos\left(\omega t\right)$, $l\bar{k}\left(t\right)=-\sqrt{2}\alpha \sin\left(\omega t\right)$, and the eigenfunction of quantum harmonic oscillator $\phi_{n}\left(x\right)=1/\left(\pi^{1/4}\sqrt{2^{n}n!}\right)H_{n}\left(x\right)\exp\left(-x^{2}/2\right)$, which carries energy $\varepsilon_{\alpha ,n}=\left(\alpha^{2}+n+1/2\right)\hbar \omega$. $H_{n}\left(x\right)$ is Hermite polynomial. Note that the corresponding wave packet oscillates in the harmonic potential (with time period 2π/ω) without changing the shape of density.

**Figure 1:**
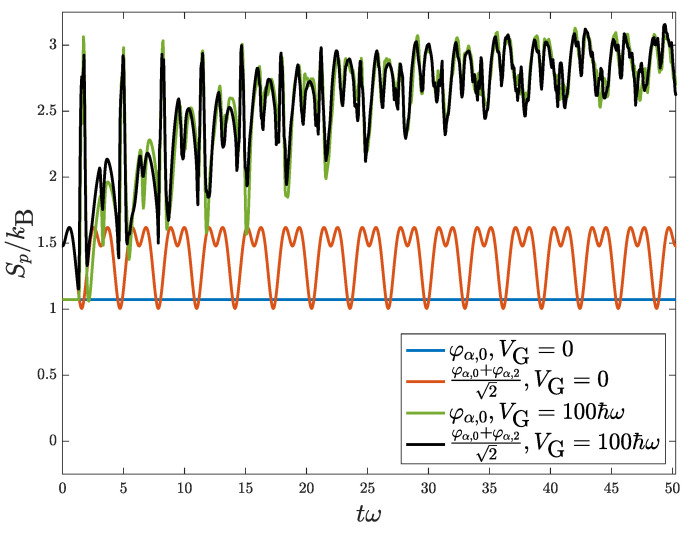
Time development of Shannon entropies for a quantum oscillator in various initial conditions. For a harmonic confining potential only, blue and red lines correspond respectively to the initial conditions with single ($\psi_{\alpha ,0}$) and the superposition of two ($\psi_{\alpha ,0}$ and $\psi_{\alpha ,2}$) GCSs. Here, we took $\alpha =15/\sqrt{2}$, which means that the initial centroid position $\bar{x}\left(0\right)$ is 15l. By the addition of a Gaussian barrier with strength $V_{G}=100\hbar \omega$ and width *l*, green and black lines correspond respectively to the initial conditions with single and the superposition of two GCSs.

The initial conditions in *x*- and *k*-space corresponding to a mixture of GCSs would have the forms

(17) $$\psi \left(x,0\right)=\sum_{n}c_{n}\varphi_{\alpha ,n}\left(x,0\right)=\frac{1}{\sqrt{l}}\sum_{n}c_{n}\phi_{n}\left[\frac{x-\bar{x}\left(0\right)}{l}\right]$$

and

(18) $$\tilde{\psi }\left(k,0\right)=\sum_{n}c_{n}{\tilde{\varphi }}_{\alpha ,n}\left(k,0\right)=\sqrt{2\pi l}\sum_{n}i^{n}c_{n}\phi_{n}\left[l(k-\bar{k}\left(0\right))\right]\exp\left[i\left(\bar{x}\left(0\right)k-\frac{\bar{x}\left(0\right)\bar{k}\left(0\right)}{2}\right)\right],$$

where $c_{n}$ is the coefficient of superposition. $\bar{x}\left(0\right)=\sqrt{2}\alpha l$ and $\bar{k}\left(0\right)=0$ are the initial centroid locations of wave packet in position and momentum spaces, respectively. Without $V_{1}$, for an initial condition with superposition of two CGSs at most, $\tilde{\psi }\left(k,t\right)=c_{n}{\tilde{\varphi }}_{\alpha ,n}\left(k,t\right)+c_{n^{\prime }}{\tilde{\varphi }}_{\alpha ,n^{\prime }}\left(k,t\right)$, $\rho_{kk}$ is read as

(19) $$\begin{array}{ccc} \frac{\rho_{kk}\left(t\right)}{2\pi l} & = & |c_{n}{|}^{2}\phi_{n}^{2}\left[l(k-\bar{k})\right]+{\left|c_{n^{\prime }}\right|}^{2}\phi_{n^{\prime }}^{2}\left[l(k-\bar{k})\right]\\ & + & 2ℜ\left[i^{\left(n-n^{\prime }\right)}c_{n}c_{n^{\prime }}^{*}\exp\left[-i\left(n-n^{\prime }\right)\omega t\right]\right]\phi_{n}\left[l(k-\bar{k})\right]\phi_{n^{\prime }}\left[l(k-\bar{k})\right], \end{array}$$

which basically still oscillates in the harmonic trap with period 2π/ω, since $\bar{k}$ is a periodic function of time, but with the shape modulated by a period $2\pi /[\left(n-n^{\prime }\right)\omega ]$ due to the factor $\exp[-i\left(n-n^{\prime }\right)\omega t]$. Here, ℜ[A] denotes the real part of *A*. For the case of single CGS, distribution Equation (19) reduces to $\rho_{kk}\left(t\right)=2\pi l\phi_{n}^{2}\left[l(k-\bar{k})\right]$, which oscillates with a period 2π/ω and without changing its shape, so that the corresponding entropy $S_{p}\left(t\right)$ is just a constant according to Equation (6) shown as the blue horizontal line in Figure 1. This can also be verified by the vanishing of the first term on the right-hand side of Equation (14) (see Appendix A for details). For the case of a mixture of GCS, the wave packet again oscillates inside the harmonic trap but with the shape of density modulated periodically. Therefore, its corresponding entropy is periodic in time as shown with the red line in Figure 1.

## 3. Barrier Potential Produces Entropy and Decoherence

As postulated earlier, a bump (barrier) potential may cause the wave scattering of an oscillating wave packet, which leads to its decoherence featured by the increasing of entropy. Here, we consider a single central Gaussian barrier, $V_{1}\left(x\right)=V_{G}\exp\left(-x^{2}/l^{2}\right)$, of which the Fourier transform is ${\tilde{V}}_{1}\left(k\right)=V_{G}l\sqrt{\pi }\exp\left(-k^{2}l^{2}/4\right)$. One can picture that, with this ${\tilde{V}}_{1}\left(k\right)$ recruited to Equation (14), it can lead to the production of entropy. Since integrating the second term into the right-hand side of Equation (14) by hand is infeasible, we can only verify this entropy production by integrating TDSE, Equation (8), numerically.

Figure 1 shows the time development of Shannon entropies for a quantum oscillator in various initial conditions. For $V=V_{0}$ only, blue and red lines correspond to the initial conditions for an off-centered single GCS ($\varphi_{\alpha ,0}$) and a mixture of two GCS’s ($\varphi_{\alpha ,0}$ and $\varphi_{\alpha ,2}$), respectively. Here, we took $\alpha =15/\sqrt{2}$, which means that the initial centroid position is $\bar{x}\left(0\right)=15l$, a position far away from the bump barrier such that the wave packet would not be immediately affected by the bump barrier right at the beginning of oscillation. The total energies of these two cases are $E=(15^{2}+1)/2\hbar \omega$ and $E=(15^{2}+3)/2\hbar \omega$, respectively. For $V=V_{0}+V_{1}$, with the recruitment of a Gaussian barrier $V_{1}$ having strength $V_{G}=100\hbar \omega$, green and black lines correspond to the initial conditions for an off-centered single GCS and a mixture of two GCS’s as above. With the increasing trend of entropy, shown by green and black lines, it verifies the entropy production owing to the inclusion of barrier potential. Note that the fluctuations of green and black lines come from the modulation of the wave packet when oscillating within the trap. As time goes on, the system reaches thermal equilibrium and the entropies of green and black lines would grow to become saturated with diminishing fluctuations. The shape of wave packet, or density distribution, at equilibrium will be discussed in the next section.

**Figure 2:**
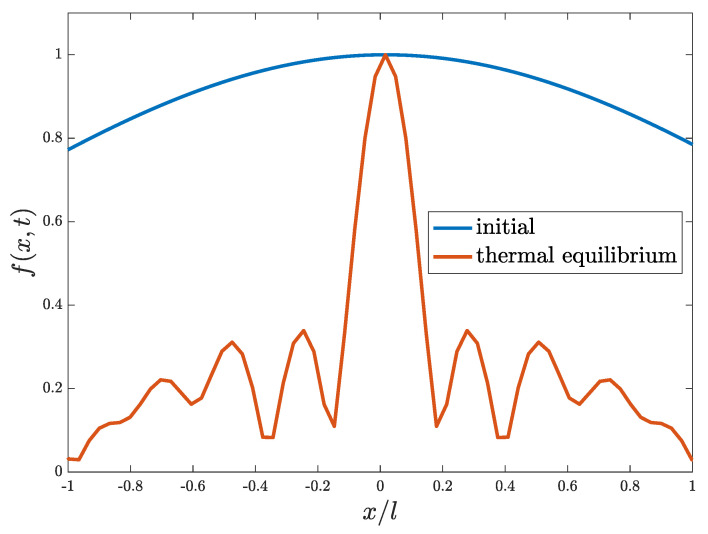
Spatial autocorrelation function plotted at *t* = 0 (blue line) and at equilibrium, $t\gg t_{\mathrm{th}}$, (red line) for the case of a single GCS ($\psi_{\alpha ,0}$), corresponding to a green line in Figure 1.

Besides producing entropy, thermalization also causes the decoherence of a wave packet. Here, we define a spatial autocorrelation function (SACF) for wavefunction in order to measure the autocorrelation length, which can serve as a good indicator of coherence in a quantum system:

(20) $$f\left(x,t\right)=\left|\int_{-\infty }^{\infty }\psi^{*}\left(x-x^{\prime },t\right)\psi \left(x^{\prime },t\right)dx^{\prime }\right|=\left|\frac{1}{2\pi }\int_{-\infty }^{\infty }\rho_{kk}\left(t\right)\exp\left(ikx\right)dk\right|.$$

f(x,t) depends intimately on the behaviors of ψ(x,t) and measures the correlation between points separated by *x* at a given time *t*. Figure 2 shows f(x,t) at an initial time and equilibrium with the blue curve corresponding to the initial one and the red curve corresponding to the equilibrium one.

At the beginning of oscillation, f(x,t=0) exhibits a close-to-unity constant distribution in space, featuring a large autocorrelation length indicating that the system is highly coherent. In contrast, when the system comes to equilibrium, $f(x,t\gg t_{\mathrm{th}})$ is largely narrowed, featuring a small autocorrelation length indicating the strong incoherence of system. $t_{\mathrm{th}}$ is the so-called thermalization time, after which the system reaches equilibrium [12]. This significant decreasing of autocorrelation length reveals the decoherence of system during thermalization.

## 4. Density Distribution When Reaching Thermal Equilibrium

**Figure 3:**
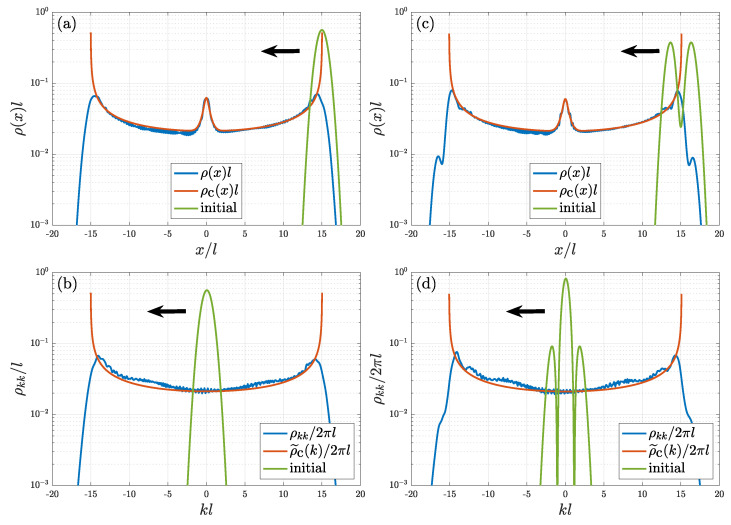
Initial (green line) and equilibrium (blue line) density distributions for a quantum oscillator in a harmonic trap with a Gaussian barrier. Red line is the best fitting from (22) and (23). Frames (**a**,**c**) correspond respectively to density distributions in position space with initial conditions $\psi \left(x,0\right)=\varphi_{\alpha ,0}$ shown in frame (**a**), and $\psi \left(x,0\right)=\left(\varphi_{\alpha ,0}+\varphi_{\alpha ,2}\right)/\sqrt{2}$ shown in frame (**c**). The counterpart density in momentum space is shown in frames (**b**,**d**). The black arrow indicates the moving direction of an initial wave packet (green line).

Figure 3 shows density distributions at initial time (green line) and equilibrium (blue line), with panels (a) and (c) in position space, and panels (b) and (d) in momentum space. One can see from panels (a) and (c), where the initial narrowly distributed ρ(x), centered at $\bar{x}\left(0\right)=15l$, evolves to a centered-at-zero, symmetric, and much broader distribution at equilibrium. Similarity for the evolution of $\rho_{kk}$ is shown in panels (b) and (d), with the initial distribution centered at 0. These distributions are very close to the microcanonical distribution for a classical harmonic oscillator. In a microcanonical ensemble of a classical harmonic oscillator with a given energy *E*, the probability distribution in phase space is

(21) $$P_{c}\left(x,k\right)=\hbar \omega \delta \left(E-V\left(x\right)-\frac{\hbar^{2}k^{2}}{2m}\right)$$

with $1/\left(2\pi \right)\int_{-\infty }^{\infty }P_{c}\left(x,k\right)dxdk=1$, where δ(⋯) is the Dirac delta function. The red lines in Figure 3 show the best fitting in position space, $\rho_{c}\left(x\right)$, and momentum space, ${\tilde{\rho }}_{c}\left(k\right)$:

(22) $$\rho_{c}\left(x\right)=\frac{1}{2\pi }\int_{-\infty }^{\infty }P_{c}\left(x,k\right)dk=\frac{m\omega }{\pi \sqrt{2m\left[E-V(x)\right]}}\;\mathrm{for}\;-x_{t}<x<x_{t},$$

and

(23) $${\tilde{\rho }}_{c}\left(k\right)=\int_{-\infty }^{\infty }P_{c}\left(x,k\right)dx≃\frac{2\hbar }{\sqrt{2mE-\hbar^{2}k^{2}}}\;\mathrm{for}-k_{t}<k<k_{t},$$

which are obtained from the microcanonical ensemble of statistical mechanics and are derived in Appendix B. Note that Equation (23) is approximated by neglecting the barrier potential $V_{1}\left(x\right)=V_{G}\exp\left(-x^{2}/l^{2}\right)$ and letting $V\left(x\right)=V_{0}\left(x\right)=m\omega^{2}x^{2}/2$. This is because, if the energy of system, *E*, is large enough, the effect of Gaussian barrier on phase-space distribution Equation (21) would be weak, and therefore the consequent distribution in momentum space can be approximated by ignoring it.

## 5. Energy Transformation

To monitor the evolution of kinetic and potential energies of the wave packet during its oscillation within the trap and the energy exchange between these two energy components, it is useful to consider the free energy of the system: $F=E-E_{0}$, where *E* is the total energy. Since total energy is conserved all the time, E(t)=E(0), with E(0) corresponding to the initial total energy of a wave packet, which is an GCS (or a mixture of GCSs) of the harmonic trap placed off-center initially. $E_{0}$ corresponds to the total energy of the initial wave packet located right at the center of trap, which would be stationary if the barrier potential $V_{1}\left(x\right)$ is neglected. Here, E=K+U and similarly $E_{0}=K_{0}+U_{0}$ with *K* ($K_{0}$) and *U* ($U_{0}$) corresponding to kinetic and potential parts of energy. Basically, $K\left(0\right)=K_{0}$ since the shape of wave packet is the same, and $U\left(0\right)>U_{0}$ due to the initial off-center displacement of the wave packet. Therefore, $F\left(0\right)=E\left(0\right)-E_{0}=U\left(0\right)-U_{0}$, which means that the initial free energy is the initial excessive potential energy. When there is no barrier potential, the wave packet would keep its coherence with free energy F(t) conserved and just re-distributed into kinetic and potential energies dynamically. It means that wave packet oscillates within the trap without thermalization as indicated by its non-increasing Shannon entropy depicted by the blue and red lines in Figure 1. Meanwhile, both $K\left(t\right)-K_{0}$ and $U\left(t\right)-U_{0}$ are periodic in time with their sum, F(t), fixed in time, which means kinetic and potential energies fully exchange with each other during oscillation similar to a harmonic oscillator free of friction.

**Figure 4:**
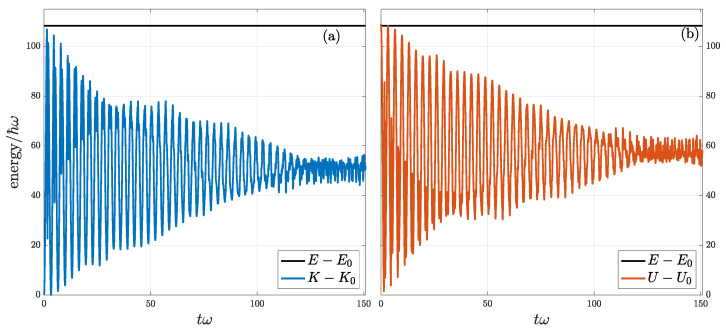
Energy evolution of a quantum harmonic oscillator with a Gaussian barrier. Frames (**a**,**b**) respectively show the evolution with time for kinetic and potential energies. Kinetic and potential energies, deducting their ground state parts, oscillate with time with diminishing amplitudes, while the total energy is still conserved. With $\psi \left(x,0\right)=\varphi_{\alpha ,0}$, the ground-state energy $E_{0}=4.70$, kinetic energy $K_{0}=0.74$, and potential energy $U_{0}=3.95$.

When barrier potential $V_{1}\left(x\right)$ exists, the oscillating wave packet is thermalized as indicated by its increasing Shannon entropy depicted by the green and black lines in Figure 1. Meanwhile, both $K\left(t\right)-K_{0}$ and $U\left(t\right)-U_{0}$ still oscillate with time but with diminishing amplitudes as shown in Figure 4. Note that their sum, F(t), is still conserved in time. This means kinetic and potential energies exchange with each other less and less during thermalization, by which we can picture the wave packet oscillating less and less within the trap due to the wave scattering by the barrier. In this sense, the barrier acts like a friction to the oscillating wave packet that transforms its initial coherent mechanical energy into an incoherent thermal one. The decoherence of wave packet during thermalization can also be observed by its density distribution getting modulated by high-frequency fluctuations, besides a large decreasing of autocorrelation length as described in Figure 2. Note that the initial excessive coherent potential energy would not be equipartitioned into incoherent $K\left(t\right)-K_{0}$ and $U\left(t\right)-U_{0}$ as shown in Figure 4 when approaching thermal equilibrium. This is because the trap is no longer a harmonic one. We can picture that, when the height of barrier is smaller, energy would be more equipartitioned and sure it would also take a longer time to reach equilibrium due to a weaker barrier.

## 6. Conclusions

In conclusion, the thermalization in an isolated oscillating BEC in a harmonic trap superposed with a Gaussian barrier is studied. We solve the dynamic wavefunction ψ(x,t) of the system and, from it, the relevant physics are obtained. The key results are: (i) The current microscopic quantum system is found to obey the macroscopic second law of thermodynamics by the increasing of Shannon entropy during thermalization, (ii) the production of entropy during thermalization is accompanied by the decoherence of system featured by the significant reduction of autocorrelation length, and (iii) the Gaussian barrier potential plays the role of dissipation with energy transformed from a coherent mechanical energy into an incoherent thermal one. (iv) At equilibrium, the density distributions both in position and momentum spaces are well fitted by a microcanonical ensemble of statistical mechanics.

## Appendix A. The Proof of a Single GCS Oscillating within a Harmonic Trap Producing No Entropy

For single GCS oscillating within a harmonic trap, the momentum-space wavefunction and its second derivative are

(A1) $$\tilde{\psi }\left(k,t\right)={\tilde{\varphi }}_{\alpha ,n}\left(k,t\right)=i^{n}\sqrt{2\pi l}\exp\left(-in\omega t\right)\exp\left[-i\left(\frac{\omega t}{2}-\bar{x}k+\frac{\bar{x}\bar{k}}{2}\right)\right]\phi_{n}\left[l(k-\bar{k})\right],$$

and

(A2) $$\partial_{k}^{2}\tilde{\psi }\left(k,t\right)=i^{n}\sqrt{2\pi l}\exp\left(-in\omega t\right)\exp\left[-i\left(\frac{\omega t}{2}-\bar{x}k+\frac{\bar{x}\bar{k}}{2}\right)\right]{\left(\partial_{k}+i\bar{x}\right)}^{2}\phi_{n}\left[l(k-\bar{k})\right],$$

since

(A3) $$ℑ\left[\tilde{\psi }\left(k,t\right)\partial_{k}^{2}\tilde{\psi }\left(k,t\right)\right]=-4\pi l\bar{x}\phi_{n}\left[l(k-\bar{k})\right]\partial_{k}\phi_{n}\left[l(k-\bar{k})\right]$$

and

(A4) $$\ln\left(\frac{\rho_{kk}}{2\pi l}\right)=\ln\left(\phi_{n}^{2}\left[l(k-\bar{k})\right]\right),$$

are odd and even functions of $\left(k-\bar{k}\right)$, respectively. Thus, the entropy production rate for single GCS in a harmonic trap vanishes:

(A5) $$\frac{dS_{p}\left(t\right)}{dt}=-\frac{k_{B}\omega }{2\pi l^{2}}\int_{-\infty }^{\infty }ℑ\left[\tilde{\psi }\left(k,t\right)\frac{\partial^{2}{\tilde{\psi }}^{*}\left(k,t\right)}{\partial k^{2}}\right]\ln\left(\frac{\rho_{kk}}{2\pi l}\right)dk=0.$$

## Appendix B. Derivation of Density Distributions in Position and Momentum Spaces at Equilibrium from Microcanonical Ensemble of Statistical Mechanics

The composition of $\delta \left(g(x)\right)$ for a continuously differentiable function *g* is defined by

(A6) $$\delta \left(g(x)\right)=\sum_{i}\frac{\delta (x-x_{i})}{|g^{\prime }\left(x_{i}\right)|},$$

where the sum extends over all roots of g(x), which are assumed to be simple. Thus, for example,

(A7) $$\delta \left(x^{2}-\alpha^{2}\right)=\frac{1}{2|\alpha |}\left[\delta (x-\alpha )+\delta (x+\alpha )\right].$$

Using the above formulas, the best fittings of thermal equilibrium density distributions in position space, Equation (22) and in momentum space, Equation (23) can be obtained as follows:

(A8) $$\begin{array}{ccc} \rho_{c}\left(x\right) & = & \frac{1}{2\pi }\int_{-\infty }^{\infty }P_{c}\left(x,k\right)dk\\ & = & \frac{\hbar \omega }{2\pi }\int_{-\infty }^{\infty }\delta \left(\frac{\hbar^{2}k^{2}}{2m}-\left[E-V(x)\right]\right)dk\\ & = & \frac{m\omega }{\pi \hbar }\int_{-\infty }^{\infty }\delta \left(k^{2}-\frac{2m}{\hbar^{2}}\left[E-V(x)\right]\right)dk\\ & = & \frac{m\omega }{\pi \sqrt{2m\left[E-V(x)\right]}}\;\mathrm{for}\;-x_{t}<x<x_{t}, \end{array}$$

and

(A9) $$\begin{array}{ccc} {\tilde{\rho }}_{c}\left(k\right) & = & \int_{-\infty }^{\infty }P_{c}\left(x,k\right)dx\\ & = & \hbar \omega \int_{-\infty }^{\infty }\delta \left(V\left(x\right)-\left(E-\frac{\hbar^{2}k^{2}}{2m}\right)\right)dx\\ & ≃ & \hbar \omega \int_{-\infty }^{\infty }\delta \left(\frac{m\omega^{2}x^{2}}{2}-\left(E-\frac{\hbar^{2}k^{2}}{2m}\right)\right)dx\\ & = & \frac{2\hbar }{m\omega }\int_{-\infty }^{\infty }\delta \left(x^{2}-\left(\frac{2mE-\hbar^{2}k^{2}}{m^{2}\omega^{2}}\right)\right)dx\\ & = & \frac{2\hbar }{\sqrt{2mE-\hbar^{2}k^{2}}}\;\mathrm{for}\;-k_{t}<k<k_{t}, \end{array}$$

with $x_{t}=\sqrt{2E/(m\omega^{2})}$ and $k_{t}=\sqrt{2mE/(\hbar^{2})}$ being the roots of *g* in position and momentum spaces when applying Equation (A6). Furthermore, using the integral formula $\int_{-\alpha }^{\alpha }dx/\sqrt{a^{2}-x^{2}}=\pi$, the normalized conditions of Equations (22) and (23) can also be obtained:

(A10) $$\begin{array}{ccc} \int_{-\infty }^{\infty }\rho_{c}\left(x\right)dx & = & \int_{-x_{t}}^{x_{t}}\frac{m\omega }{\pi \sqrt{2m\left[E-V(x)\right]}}dx\\ & ≃ & \int_{-x_{t}}^{x_{t}}\frac{1}{\pi \sqrt{x_{t}^{2}-x^{2}}}dx\\ & = & 1, \end{array}$$

and

(A11) $$\begin{array}{ccc} \int_{-\infty }^{\infty }{\tilde{\rho }}_{c}\left(k\right)dk & = & \int_{-k_{t}}^{k_{t}}\frac{2\hbar }{\sqrt{2mE-\hbar^{2}k^{2}}}dk\\ & = & \int_{-k_{t}}^{k_{t}}\frac{2}{\sqrt{k_{t}^{2}-k^{2}}}dk\\ & = & 2\pi . \end{array}$$
